# Erie County Medical Society and Preliminary Education

**Published:** 1875-05

**Authors:** 


### Erie County Medical Society and Preliminary Education.

Below we present our readers with a letter taking exception to some of the statements in a communication to the *Medical Record* of May 22d.

The charges against the member of the Society mentioned in that letter, were preferred, as many of our readers are aware, against the Senior Editor of this JOURNAL, such being the case we have thus far refrained from saying any thing in regard to the matter, and our correspondent has presented our views so fully that we have nothing to add. The remarks which we have already made under the head of "The American Medical Association and Medical Education," will sufficiently explain our views upon the general subject. We are, however, surprised that the correspondent of the *Record* should allow the impression to be made that the charges preferred at the January meeting resulted in anything like a resolution of censure, partaking as he says, of a character of "*diplomatic indefiniteness.*" He must have known that they were wholly dismissed, the simple addition to the resolution being that the Society committed itself to the advisability of preliminary examination of medical students. We shall welcome the time when the qualifications suitable to the commencement of the study of medicine shall be definitely settled and some plan established of determing those qualifications. It seems to us that in their determination the moral as well as the mental qualifications should have some weight.

The preliminary examination should be undertaken by the Colleges, and we are glad to see indications of a move in that direction, when such a step is taken and the system firmly established, we may look for a real advance in the standard of medical education. E. B.

---

*To the Editor of Buffalo Medical and Surgical Journal.*

DEAR SIR:—In the *Medical Record* of May 22d, which reached me this morning, I find some statements in regard to the Erie County Medical Society and Preliminary Education which seem to demand a little attention. In the outset do not understand me to convey the idea by any portion of this letter that preliminary education of medical students should not be insisted upon, on the contrary, I am heartily in favor of the most thorough preliminary education, and am willing to lend my aid to any move which shall strive to bring about this desired reform. The correspondent of the *Record* says that for four years there has been a systematic and sustained effort in the Erie County Medical Society to faithfully execute the by-laws in regard to this matter, and further states that the move which finally aroused the Society was taken at the annual meeting in January last. This move was the preferment of charges against "a prominent member of our Faculty and Society," for "knowing repeated and willful" violation of the by-laws in admitting students to his office without the required certificate of preliminary examination. To the members of the Erie County Medical Society who were present at the January meeting the facts are doubtless sufficiently fresh not to demand a repetition here, suffice it to say, that the prominent member aforesaid was wholly exonorated from each and every charge, by the adoption of a resolution, which, however, was not of that *indefinite* character that the correspondent of the *Record* would have us believe, neither was it in *any sense*, indefinite or otherwise, a resolution of censure.

In common with other members of the Society the question suggested itself to me, why did the primary board select only one, or the one whom they did, against whom to prefer charges. They must certainly have known that there were students of medicine in the offices of many other members of the Erie County Medical Society who had never presented themselves to the Board for examination, and it seems to me that one-half the labor expended in the unsuccessful endeavor to secure affidavits, etc., might have proven such to be the fact. Of the motive of these charges, however, I have nothing to say. The member against whom they were preferred has seen fit to let the matter drop, and I do not propose to again open the case.

One single statement in the letter demands further notice and I have done. The correspondent carries the idea that students who have commenced the study of medicine without preliminary examination have done so in an illegal way, and that diplomas granted to them have been granted *contrary to law*, and do not entitle the holders to all the rights and privileges of the practice of medicine. I have yet to learn that the *law* has anything to do with the preliminary education of medical students. The requirement is one made of their preceptors and does not in any way effect them, moreover it is simply a requirement of medical societies, and the law, as I read it, does not give these societies any power to regulate the practice of medicine. If a young man sees fit to graduate without a preliminary examination, his diploma confers upon him *all the rights and previleges* that it ever did.

Nothing is ever gained in any cause by misrepresentation. The resolution as it is, is sufficiently binding and definite in its requirements, but if the chairman of the Primary Board attempts to add a legal force *which it does not possess*, he will convey the idea that he is striving to defend a cause which can not stand on its own merits.

A MEMBER OF THE ERIE COUNTY MEDICAL SOCIETY.

May 24, 1875.

